# A colloidal gold immunochromatographic test strip based on mAbs anti-N protein to detect feline coronavirus

**DOI:** 10.1128/spectrum.01830-24

**Published:** 2025-06-02

**Authors:** Zhe Liu, Yupeng Yang, Ruibin Qi, Haorong Gu, Mengru Chen, Kexin Feng, Qian Jiang, Honglin Jia, Hongtao Kang, Jiasen Liu

**Affiliations:** 1 State Key Laboratory for Animal Disease Control and Prevention, Harbin Veterinary Research Institute, Chinese Academy of Agricultural Sciences, Harbin, China; 2 College of Veterinary Medicine, Northeast Agricultural University 12430, Harbin, China; Luciana Jesus Costa, Universidade Federal do Rio de Janeiro, Rio de Janeiro, Rio de Janeiro, Brazil

**Keywords:** feline coronavirus (FCoV), monoclonal antibody, identification of antigenic epitopes, colloidal gold immunochromatographic text strip

## Abstract

Feline coronavirus (FCoV) includes Feline enteric coronavirus (FECV) and feline infectious peritonitis virus (FIPV). FECV primarily affects the gastrointestinal system of cats, causing mild and self-limiting gastrointestinal symptoms. In contrast, FIPV infection leads to severe immune-mediated disease with rapid progression and a very high mortality rate. The occurrence of feline infectious peritonitis (FIP) is associated with mutations in the viral genome during FECV infection, which makes diagnosing FECV an effective measure for preventing FIP. This study aims to establish rapid and efficient detection methods to provide reliable diagnostic tools for disease prevention and treatment. In this study, the recombinant plasmid pCold I-FIPV-DF-2-N of the FIPV-DF-2 virus N protein was constructed. It was then expressed prokaryotically and purified. Using this plasmid, BALB/c mice were immunized, and two highly specific and stable monoclonal antibodies, mAbs 2G7 and mAb 3H5, were successfully prepared. mAb 2G7 recognizes the amino acid sequence 18RGRSNSRGRKN28 on the N protein, and mAb 3H5 recognizes the amino acid sequence 295GDQVKVTLTHTYYLPKDDAKTSQFLEQID323. We successfully developed a colloidal gold immunochromatographic test strip for FCoV detection using a double-antibody sandwich method. Experimental results showed that this test strip had a detection limit of 209.8 ng/mL for the recombinant pCold I-FIPV-DF-2-N protein; the overall agreement rate for detecting FCoV in 10 ascites samples, which tested positive by conventional PCR, was 70%; and there was no cross-reaction with feline parvovirus, feline calicivirus, and feline herpesvirus. The detection method can realize the rapid detection on site and provide a convenient detection method for pet clinics, animal shelters, etc.

**IMPORTANCE:**

In recent years, pets have become an indispensable part of people’s lives. As companion animals, the number of pet cats in domestic families has increased year by year. According to the 2023 Pet White Paper, the number of pet cats in China has reached more than 65 million. Therefore, the prevention and treatment of infectious diseases in cats is becoming more and more important. Therefore, in this study, a double-antigen sandwich colloidal gold immunochromatographic assay was developed to detect feline coronavirus (FCoV). The detection of feline enteric coronavirus (FECV) excreted by cats can effectively implement treatment, reduce the coronavirus load, and further prevent the risk of FECV infection evolving into feline infectious peritonitis virus. This method is simple, rapid, and specific and can be used for the detection of FCoV. Therefore, this method is more suitable for pathogen diagnosis in the field than previous methods.

## INTRODUCTION

Feline coronavirus (FCoV) is a positive-strand RNA virus that belongs to the family Coronaviridae and the genus *Alphacoronavirus* (1). It is commonly found in both domesticated and wild felines. Based on distinctive clinical symptoms and pathological alterations, FCoV can be divided into two biotypes: Feline infectious peritonitis virus (FIPV) and feline enteric coronavirus (FECV) (2). FECV is highly prevalent in the feline intestinal tract, and infected cats typically exhibit mild gastrointestinal symptoms that resolve spontaneously, resulting in low mortality rates (2). In contrast, FIPV infection leads to a severe immune-mediated disease characterized by fibrin or granulomatous serositis, with a high fatality rate and rapid progression (3). It has been demonstrated that feline infectious peritonitis (FIP) arises from mutations occurring within the viral genome during FECV infection (4). Despite its relatively low incidence among FECV-infected cats (5), FIP remains a significant contributor to feline mortality. The current diagnostic approach for FIP primarily relies on retrospective disease history surveys, common hematological experiments, and other auxiliary diagnostic methods due to the lack of accurate and reliable diagnosis of FIP (6). Therefore, precise diagnosis of FCoV is crucial for the management and control of FIPV.

The genome length of FCoV is approximately 29 kb, containing various functional regions such as open reading frames (ORFs) and untranslated regions. Some important ORFs include ORF1a and ORF1b, which encode RNA-dependent RNA polymerase and other non-structural proteins (7). They contain important regulatory sequences that initiate the transcription and translation of viral genes. ORF3 is located between the S and E genes, encoding proteins 3a, 3b, and 3c, which are involved in virus assembly and release (8, 9). ORF7 is located downstream of the N gene, encoding proteins 7a and 7b, which may participate in the interaction between the virus and the host as well as the pathogenic mechanism (10–12). The composition of FCoV virions, similar to other coronaviruses, primarily comprises nucleocapsid (N), envelope (E), membrane (M), and spike (S) proteins (11). The S protein is considered a viral regulatory factor for binding and entry into cells, while also participating in the tropism and virulence of FCoV, as well as the transition from enteric disease to FIP (13). While the S protein functions as a neutralizing antigen for FCoV and elicits antibodies with neutralizing activity, its high variability and presence of Antibody-Dependent Enhancement (ADE) epitopes make it unsuitable for detection purposes (14–16). Conversely, the N protein plays a pivotal role in viral replication, assembly, and immunity as the principal structural protein in FCoV. Extensive studies have demonstrated its strong immunogenicity and ability to induce robust cellular and humoral immunity (17–20). Moreover, compared to the spike protein, the N gene exhibits significant conservation within coronaviruses, making it an excellent target for both antigen and antibody detection (21). In the early stages of FCoV infection, the cat can produce high levels of antibodies against the N protein (22, 23). As a result, the N protein can serve as a crucial indicator for prompt and early detection of FCoV infection. Currently, there is a lack of rapid diagnostic products for FCoV; only RT-PCR can be utilized for swift detection. However, conventional pet hospitals do not possess corresponding detection equipment, which significantly impedes disease detection, diagnosis, and treatment. Therefore, providing an expedient, convenient, and cost-effective method for early rapid detection becomes an urgent concern in this field.

## MATERIALS AND METHODS

### Virus strains, cells, animals, and main reagents

The FIPV strain DF-2 (GenBank accession number: JQ408981.1), feline parvovirus (FPV) strain XJ-1 (GenBank accession number: EF988660.1), the feline herpesvirus (FHV) strain HRB2019 (GenBank accession number: PP034747), and the feline calicivirus (FCV) strain FCV-2280 (GenBank accession number: KC835209.1), CRFK, 293T and SP2/0 myeloma cells were stored in our laboratory. The BALB/c mice were purchased from Beijing Vital River Laboratory Animal Technology Co., Ltd. QuickAntibody-Mouse 5W was purchased from Beijing Biodragon Immunotechnologies Co., Ltd. The hypoxanthine, aminopterin, and thymidine supplement (HAT), the hypoxanthine and thymidine supplement, and polyethylene glycol solution (PEG) were obtained from Sigma Aldrich Co., Ltd. HisSep Ni-NTA Agarose Resin was purchased from Yeasen Biotechnology (Shanghai) Co., Ltd.

### Preparation of FIPV-DF-2-N recombinant protein

#### Cloning of the recombinant FIPV-DF-2-N gene

The genomic sequence encoding the FIPV-DF-2-N protein (GenBank accession number: JQ408981.1) was downloaded from NCBI and cloned into the pCold I expression vector. *Bam*H I and *Hin*d III were selected as the dual restriction enzyme sites. Specific primers 5′-ctcggtaccctcgagggatccATGGCCACACAGGGACAACG-3′ (F) and 5′- agactgcaggtcgacaagcttTTAGTTCGTAACCTCATCAATCATCTC-3′ (R) were designed and sent to Comate Bioscience Co., Ltd. for synthesis. The RNA of FIPV-DF-2 was extracted using the FineQuick viral RNA extraction kit (JiFan, Beijing, China). The reverse transcription reaction was performed using the HiScript III 1st Strand cDNA Synthesis Kit (Vazyme, Nanjing, China) to synthesize the first-strand cDNA of FIPV-DF-2. Subsequently, FIPV-DF-2-N gene fragments were amplified by PCR using the obtained cDNA. The resulting PCR products were purified and retrieved utilizing a DNA Gel Extraction Kit (Omega, China). Finally, the retrieved fragments were cloned into the pCold I vector (TaKaRa, China), which was named pCold I-FIPV-DF-2-N. The recombinant plasmid was sequenced to confirm the successful integration.

### Expression and purification of the recombinant FIPV-DF-2-N protein

We transformed the recombinant plasmid into *Escherichia coli* strain BL21(DE3) and cultured the bacteria in Luria-Bertani (LB) liquid medium supplemented with 0.5 mM isopropyl β-D-1-thiogalactoside (IPTG). The culture was incubated at 16°C for 18 hours with shaking at 150 rpm. Soluble products were purified using HisSep Ni-NTA Agarose Resin (Yeasen Biotechnology, Shanghai), followed by analysis of fusion protein expression through SDS-PAGE. The purified protein was analyzed by Western blot using a specific antibody against the His-tagged protein, and the protein concentration was determined using the bicinchoninic acid (BCA) protein assay kit.

### Preparation of monoclonal antibodies against FCoV‐DF2‐N

We mixed the purified N protein antigen (20 µg/dose) with QuickAntibody-Mouse 5W adjuvant and intramuscularly immunized 6–8-week-old female BALB/c mice (*n* = X) following standard protocols. A booster dose was given on day 21, and a total of two doses was given. The antibody titers were determined by the indirect immunofluorescence assay (IFA) method on the 35th day. If the serum of the mice fails to reach the ideal titer of 1:1,280 during the study, the inoculation will be continued. The mice that have reached the desired titer were selected for the isolation of antibody-secreting lymphocytes. Splenic lymphocytes obtained from these immunized mice were then fused with mouse myeloma cells (SP2/0) at a ratio of 10:1 using a PEG reagent as fusing agent. The hybridoma cells were then cultured in HAT medium. The lack of hypoxanthine-guanine phosphoribosyltransferase (HGPRT) gene in myeloma cells made them sensitive to HAT medium and unable to survive. Once the survival hybridoma cell mass reached more than one-third of each well, their supernatants were assessed using indirect IFA. The hybridoma cells with positive supernatant were individually injected into 96-well plates using the MA900 Multipurpose Cell Sorter (Sony, Japan). In the 96-well plate, only one cell was injected into each well for subsequent cloning and cultivation. An appropriate amount of culture medium and hybridoma feeder additive factors (Biodragon, Beijing, China) was added to enable each cell to proliferate and form a clone. After a period of incubation, the growth of cells in each well was examined, and hybridoma cells that stably secreted mAbs were obtained. BALB/c multiparous female mice were pre-stimulated with 500 µL special adjuvant (Biodragon, Beijing, China) 1 week before the collection of ascites. Subsequently, these mice were intraperitoneally injected with 500 µL of hybridoma cells (10^6^ cells per mouse) suspended in phosphate-buffered saline (PBS). The ascites fluid collected from the intraperitoneal cavity of the mice was then purified using the protein G method.

### Characterization of monoclonal antibodies against FIPV-DF-2-N protein

#### Functional verification of monoclonal antibodies

The reactogenicity and specificity of the monoclonal antibody were determined by immunoblot analysis using the recombinant protein FIPV-DF-2-N and the FPV, FCV, and FHV whole-virus proteins that were purified through sucrose gradient density centrifugation. The specific binding of the monoclonal antibody to the naturally infected virus was detected by IFA.

#### Determination of the minimal antigenic epitope

Primers were designed using Oligo 7 (Table S1), and the restriction endonuclease sites of *Sfi* I and *EcoR* I were added to the upstream primer and the downstream primer, respectively. The truncated gene fragments were ligated to pCMV-HA-DsRed vectors and then expressed as fusion proteins with DsRed-tags. To further validate the length of the epitope, we transfected the pCMV-HA-DsRed recombinant plasmid containing the truncated gene fragment into 293T cells, resulting in the expression of a DsRed-tagged protein. Subsequently, these recombinant proteins were identified using mAbs, and their authenticity was further confirmed by Western blot.

#### Homology analysis for the minimal epitope

The antigenic epitope of the monoclonal antibody was compared with the corresponding target regions of 45 strains of FCoV viruses published in the NCBI database (Table 1) using MegAlign software and WebLogo online tool. This analysis aimed to evaluate the conservativeness of the antigenic epitope of the monoclonal antibody.

**TABLE 1:** The GenBank accession numbers of FCoV and FPV virus strain sequences were retrieved from NCBI

Name of strain	GenBank
FCoV strains obtained from NCBI	MN165107, KY566211, KY566210, KY566209, KY292377, KX722529, KF530123, JN183883, JN183882, HQ392472, HQ392471, HQ392470, HQ392469, HQ012372, HQ012371, HQ012370, HQ012369, HQ012368, HQ012367, GU553362, GU553361, FJ938062, FJ938061, FJ938060, FJ938059, FJ938058, FJ938057, FJ938056, FJ938055, FJ938054, FJ938054, FJ938052, FJ938051, DQ010921, DQ286389, NC_002306.3, MW030109, MT239439, KC461236, KC461235, JN634064, AY994055, JQ408981, JQ408980, GQ152141

### Development and assessment of immunochromatographic test strips

#### The preparation of the immunochromatographic strips

In order to ensure the quality and efficiency of colloidal gold immunochromatographic test strips, we chose Shenzhen Zhenrui Biotechnology Co., Ltd. to undertake the preparation. The colloidal gold test strip was prepared using the double-antibody sandwich method. mAb 3H5 was conjugated with gold nanoparticles, and the test line was coated with specific antibody mAb 2G7. The quality control line was coated with a specific goat anti-mouse IgG antibody.

#### Specificity and sensitivity of the immunochromatographic strips

The sensitivity of the immunochromatographic strip was evaluated with serial dilutions of the recombinant pCold I-FIPV-DF-2-N protein. The lowest concentration at which the protein can be detected is defined as the sensitivity of the immunochromatographic strip. The samples for the specificity test were selected from common feline viruses obtained from our laboratory.

#### Clinical sample detection of the immunochromatographic strips

Clinical samples obtained from 10 feline ascites samples were analyzed by the colloidal gold immunochromatographic strip and PCR methods (24). By comparing the results from both the immunochromatographic test strip and PCR, we can ensure the accuracy and reliability of our findings.

## RESULTS

### Cloning, expression, and purification of the recombinant FIPV-DF-2-N protein

**Fig 1:**
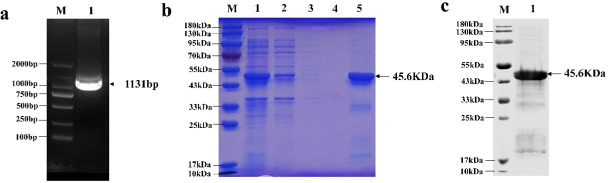
Expression and purification of FIPV-DF-2-N gene. (a) Identification of the amplified products. 1: amplified products of FIPV-DF-2-N gene; M: DL2000 DNA Marker. (b) SDS-PAGE (M: protein marker; 1: induced bacterial supernatant; 2: induced bacterial precipitation; 3: uninduced bacterial supernatant; 4: uninduced bacterial precipitation; 5: expression and purified products of FIPV-DF-2-N). (c) Western blot: (M: protein marker; 1: expression and purified products of FIPV-DF-2-N).

The gene fragments of FIPV-DF-2-N were amplified by PCR, and the results were purified and retrieved utilizing a DNA Gel Extraction Kit (Fig. 1a). Subsequently, the retrieved fragments were cloned into the pCold I vector, and the successful integration sequence was validated by Sanger sequencing. The confirmed recombinant plasmid was transformed into BL21 DE3 and cultured in LB medium with 0.5 mM IPTG for 18 hours at 16°C, with shaking at 150 revolutions per minute. The expression of the fusion protein was analyzed through SDS-PAGE (Fig. 1b). Subsequently, purified proteins were subjected to Western blot analysis using antibodies specific to the His-tagged proteins (Fig. 1c), which showed that the molecular weight of this protein was approximately 45.6 kDa.

### Preparation of monoclonal antibodies against FCoV‐DF2‐N

**Fig 2:**
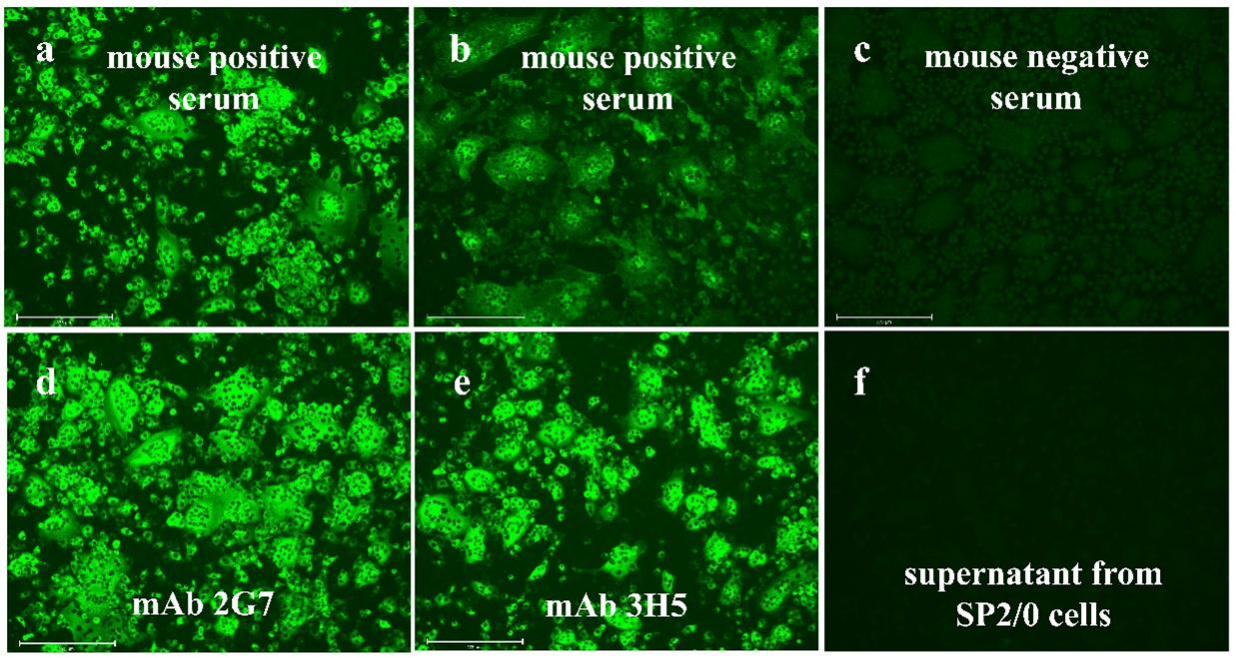
Immunogenicity of mouse positive serum and monoclonal antibodies 2G7 and 3H5 raised against FIPV-DF-2-N to virus detection. (a) IFA results of mouse positive serum (diluted 1:64) in CRFK cells infected with FIPV-DF-2; (b) IFA results of mouse positive serum (diluted 1:1,280) in CRFK cells infected with FIPV-DF-2; (c) IFA results of mouse negative serum in CRFK cells infected with FIPV-DF-2; (d) IFA results of mAb 2G7 in CRFK cells infected with FIPV-DF-2; (e) IFA results of mAb 3H5 in CRFK cells infected with FIPV-DF-2; (f) IFA results of the supernatant from SP2/0 cells in CRFK cells infected with FIPV-DF-2; for all panels, 100 TCID_50_ of FIPV-DF-2 were used for infecting the CRFK cells.

IFA detection method was used to detect the antibody titer of the serum from mice immunized twice. Clear fluorescence was still observed after 1:1,280 serial dilution of the serum, indicating a good immune effect (Fig. 2a through c). The mice that achieved the desired titer were selected for the isolation of antibody-secreting lymphocytes. Splenic lymphocytes obtained from these immunized mice were then fused with mouse myeloma cells (SP2/0) at a ratio of 10:1 using a PEG reagent as fusing agent. The hybridoma cells were then cultured in HAT medium. The lack of the HGPRT gene in myeloma cells made them sensitive to HAT medium and unable to survive. Once the survival hybridoma cell mass reached more than one-third of each well, their supernatants were assessed using indirect IFA. Hybridoma cells with positive supernatants were used with the MA900 Multi-Application Cell Sorter (Sony, Japan) and individually injected into 96-well plates. Only one cell was injected into each well for subsequent cloning and culture. In a 96-well plate, an appropriate amount of medium and hybridoma feeder additive factors (Biodragon, Beijing, China) was added, and each cell was allowed to proliferate to form a clone. After a period of incubation, mAbs 2G7 and 3H5, specifically targeting the N protein of FIPV-DF-2, were obtained (Fig. 2c through e). The specific steps of the IFA experimental method are as follows: CRFK (Crandall-Reese feline kidney) cells are inoculated with FIPV-DF-2 virus at a dose of 100 tissue culture infectious dose (TCID_50_). Mouse positive serum or hybridoma cell supernatants are then added for detection. Subsequently, Alexa Fluor 488-labeled secondary antibodies are bound to the samples. After incubation, the results are observed under a fluorescence microscope.

### Specificity of monoclonal antibodies

**Fig 3:**
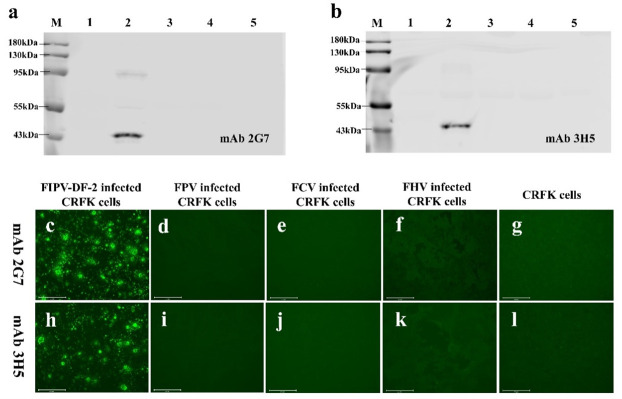
Specificity of monoclonal antibodies 2G7 and 3H5 raised against FIPV-DF-2-N to virus detection. (**a, b**) Western blot: (M: protein marker; 1: CRFK cell lysates; 2: FIPV-DF-2; 3: FPV; 4: FCV; 5: FHV); (**c through f**) IFA results of mAb 2G7 in CRFK cells infected with FIPV-DF-2, FPV, FCV, and FHV; (g) IFA results of mAb 2G7 in virus-free CRFK cells; (**h through k**) IFA results of mAb 3H5 in CRFK cells infected with FIPV-DF-2, FPV, FCV, and FHV; (l) IFA results of mAb 3H5 in virus-free CRFK cells.

To ascertain the specificity of the monoclonal antibodies (mAbs 2G7 and 3H5), we analyzed their reactivity against whole viral proteins of FIPV-DF-2, feline parvovirus (FPV), feline calicivirus (FCV), and feline herpesvirus (FHV-1) by Western blotting. The result showed that both mAbs 2G7 and 3H5 exhibited reactivity toward the N protein and purified whole viral protein of FIPV-DF-2 (45.6 kDa), while no reactivity was observed with the other three purified FPV, FCV, and FHV (Fig. 3a and b)

To further analyze the reactivity of mAbs 2G7 and 3H5 with FIPV-DF-2, we performed indirect IFA using FIPV-DF-2-infected CRFK cells. In the experimental group, CRFK cells were inoculated with FIPV-DF-2 to test the specificity of mAbs 2G7 and 3H5. The control group included uninoculated CRFK cells (negative control) and cells inoculated with FPV, FCV, or FHV (specificity control). After viral infection, the cells were fixed with ice-cold methanol, incubated with the mAbs, and then bound with Alexa Fluor 488-labeled secondary antibody. Finally, the results were observed under a fluorescence microscope to assess the specificity of the mAbs.

The IFA results demonstrated that distinct green fluorescence was observed in CRFK cells inoculated with FIPV-DF-2 (Fig. 3c and h). No green fluorescence was detected in the negative controls or in CRFK cells infected with FPV, FCV, or FHV (Fig. 3d through g and 3i through l). These findings indicate that mAbs 2G7 and 3H5 can specifically target FIPV-DF-2 antigens and possess significant reactivity.

### Determination of the minimal antigenic epitope

**Fig 4:**
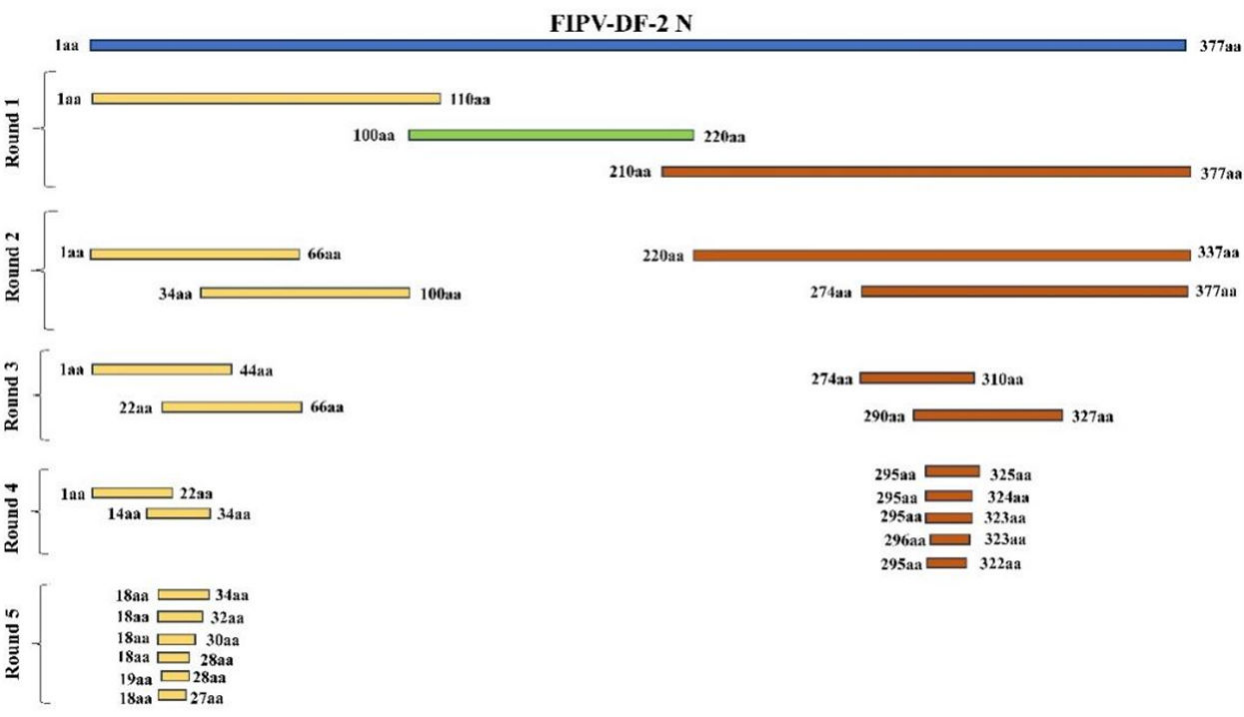
Truncated expression for Mab epitope mapping. Schematic representation of FIPV-DF-2-N fragments used for B cell epitope mapping. The original whole N (377 aa) was marked with blue. The B cell epitopes are achieved through a series of overlapping truncated N fragments, which are distinguished by the colors yellow, green, and red. Five rounds of N peptides were conducted to investigate the epitopes of the generated mAb 2G7, and four rounds of N peptides were conducted to investigate the epitopes of the generated mAb 3H5.

**Fig 5:**
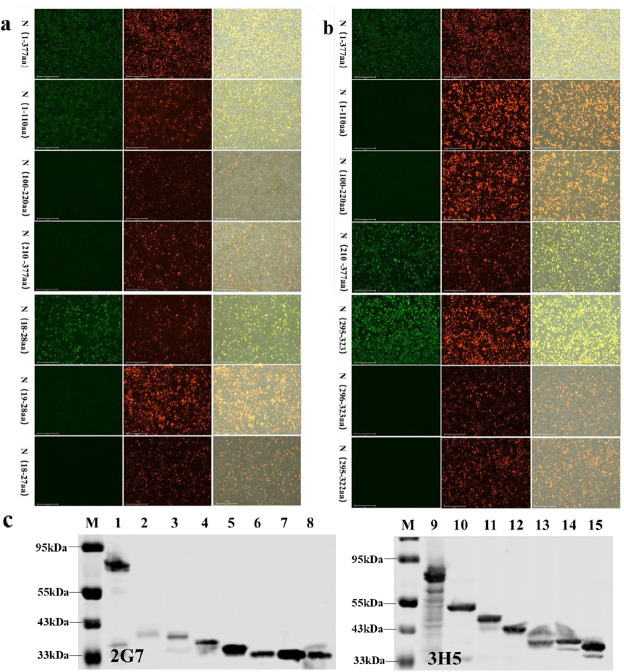
Minimal antigenic epitope determination and their specificities. Red fluorescence: represents the expression of truncated N gene fragments. Green fluorescence: represents the specific recognition of epitopes within the N gene by mAbs. Red/green merged: displays the location of truncated N proteins specifically recognized by the antibody. (a) IFA identification of mAb 2G7 epitopes; (b) IFA identification of mAb 3H5 epitopes; (c) the epitopes of mAbs 2G7 and mAb 3H5 were identified by Western blot: M: protein marker; 1: N protein (1–377 aa); 2: peptide (1–110 aa); 3: peptide (1–66 aa); 4: peptide (1–44 aa); 5: peptide (14–34 aa); 6: peptide (18–32 aa); 7: peptide (18–30 aa); 8: peptide (18–28 aa); 9: peptide (19–28 aa); 10: peptide (18–27 aa); 11: N protein (1–377 aa); 12: peptide (210–377 aa); 13: peptide (220–337 aa); 14: peptide (274–377 aa); 15: peptide (295–325 aa); 16: peptide (295–324 aa); 17: peptide (295–323 aa); 18: peptide (296–323 aa); 19: peptide (295–322 aa).

To identify the antigenic epitopes of mAbs 2G7 and 3H5, three overlapping truncated fragments, N (1–110 aa), N (100–220 aa), and N (210–377 aa), were cloned into the pCMV-HA-DsRed vector and expressed as fusion proteins tagged with DsRed in 293T cells. The full-length N (1–377 aa) protein was used as a control. IFA analysis revealed that mAb 2G7 was able to recognize N (1–110 aa) and the entire N protein, while N (100–220 aa) or N (210–377 aa) were not recognized. Conversely, fragment N (210–377 aa) and the entire N protein were recognized by mAb 3H5, while N (1–110 aa) or N (100–220 aa) were not. These results indicated that the antigenic epitopes of mAbs 2G7 and 3H5 were preliminarily determined to be located within N (1–110 aa) and N (210–377 aa), respectively. Based on Fig. 4, further truncation experiments were conducted by expressing truncated gene fragments of N (1–110 aa) and N (210–377 aa). These truncated gene fragments were fused with the pCMV-HA-DsRed vector to obtain recombinant plasmids. These plasmids were then transfected into 293T cells for expression as DsRed-tagged proteins, and IFA was used for identification. IFA analysis showed that the sequence 18 RGRSNSRGRKN 28 was the minimal linear epitope for mAb 2G7 (Fig. 5a), while 295 GDQVKVTLTHTYYLPKDDAKTSQFLEQID 323 was the minimal linear epitope for mAb 3H5 (Fig. 5B). Western blot analysis of the series of recombinant proteins obtained confirmed these findings (Fig. 5c).

### Homology analysis for the minimal epitope

**Fig 6:**
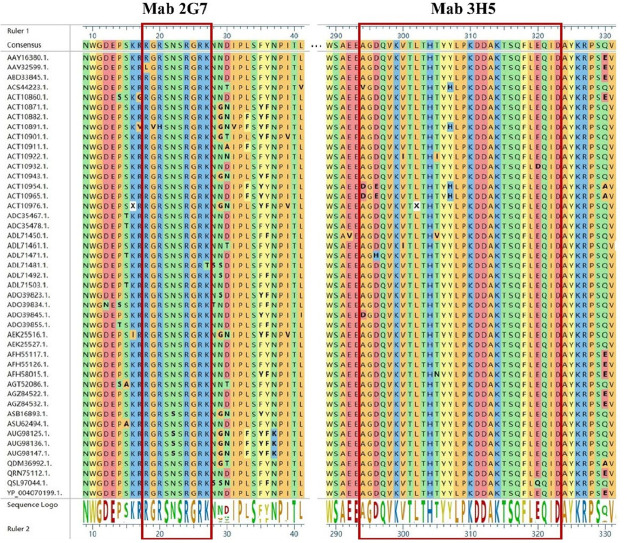
Epitope conservation analysis of mAbs 2G7 and mAb 3H5. Epitope conservation analysis of mAbs 2G7 and mAb 3H5. The epitope recognized by mAb 2G7 had a complete homology of 97.78% with the virus strain sequences, and the epitope recognized by mAb 3H5 had a complete homology of 98.62% with the virus strain sequences.

Using MegAlign software and the WebLogo online tool, we compared the antigen epitope of the monoclonal antibody with the corresponding target regions of 45 strains of FCoV virus (Table 1), which were already published in the NCBI database. This analysis aimed to assess the conservativeness of the monoclonal antibody antigen epitope. To confirm the conservation of the linear epitope recognized by mAbs 2G7 and 3H5, related 45 complete genome sequences of FCoV were aligned. The result in Fig. 6 showed that the epitope recognized by mAb 2G7 had a complete homology of 97.78% with the virus strain sequences, and the epitope recognized by mAb 3H5 had a complete homology of 98.62% with the virus strain sequences. Among the 45 FCoV complete genome sequences, six strains of FCoV exhibited single-base mutations and double-base mutations in two strains in the epitope recognized by mAb 2G7. In contrast, within the same 45 virus strain sequences, the epitope recognized by mAb 3H5 displayed single-base mutations in eight strains of FCoV and double-base mutations in three strains. Fewer mutations generally indicate a more conserved epitope. Therefore, mAbs 2G7 and 3H5 are capable of broadly and effectively recognizing FCoV, providing robust support for their stability and reliability in practical applications.

### The preparation and evaluation of the immunochromatographic strips

**Fig 7:**
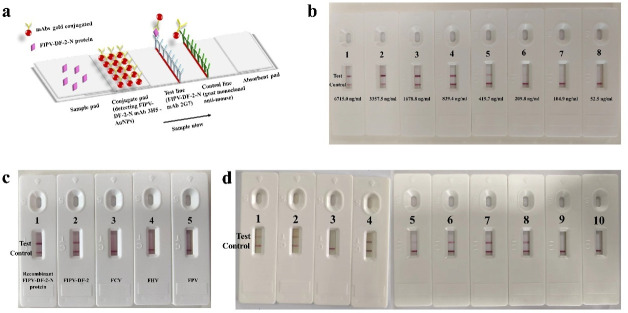
Development and evaluation of immunochromatographic test strips. (a) Schematic diagram of double-antibody sandwich colloidal gold immunochromatographic strip for detection. Validity: the distinct coloration observed on the control line serves as a confirmation of the functionality and reliability of the colloidal gold immunochromatographic test strip. Positive result: The emergence of a prominent and distinct color band on the test line. Negative result: The absence of visible coloration on the test line. (b) Sensitivity evaluation of immunochromatographic strips. The sensitivity of the immunochromatographic assay was evaluated by serial dilution of the recombinant pCold I-FIPV-DF-2-N protein. The detection limit for sensitivity was determined to be 209.8 ng/mL. (c) Specificity evaluation of immunochromatographic strips. (d) The results of the clinical samples (feline ascites). These are the test results of 10 clinical samples of cat ascites collected from a pet clinic in Harbin. Positive results: (1, 2, 4, 5, 6, 7, 8); negative results: (3, 9, 10).

If there is a substance to be detected in the sample, it first binds to the colloidal gold-labeled antibody and then migrates to the detection line through capillary action, where it binds to the detection line antibody, forming a double-antibody sandwich structure consisting of the colloidal gold-labeled antibody, the antigen to be detected, and the detection line antibody (Fig. 7a).

To evaluate the sensitivity of the recombinant pCold Ⅰ-FIPV-DF-2-N protein, we performed a dilution series assay. The protein was serially diluted in a twofold gradient with the PBS solution to concentrations of 6715.0 ng/mL, 3357.5 ng/mL, 1678.8 ng/mL, 839.4 ng/mL, 419.7 ng/mL, 209.8 ng/mL, 104.9 ng/mL, and 52.5 ng/mL and detected using the immunochromatographic strip. When the detection concentration reached 209.8 ng/mL, a relatively clear test line could still be observed on the immunochromatographic strip (Fig. 7b). This indicates that the test strips prepared in this experiment have good sensitivity.

The specificity test results showed no cross-reaction when detecting FPV, FCV, and FHV using the immunochromatographic strips, demonstrating good specificity (Fig. 7c). These results suggest that the immunochromatographic strips exhibited good reactivity and specificity in detecting FCoV.

### Clinical sample detection of the immunochromatographic strips

The study employed a colloidal gold immunochromatographic test strip to accurately detect 10 clinical samples of feline ascites, with the results further confirmed by conventional PCR (24). During the test strip detection, seven samples displayed a positive reaction, exhibiting a distinct red change at the detection line, indicative of the presence of the target antigen. Conversely, the remaining three samples exhibited a negative reaction, with no observable color change, suggesting the absence of the target antigen (Fig. 7d). Although the test strip provided an initial basis for judgment, PCR was utilized to ensure the accuracy of the findings. The PCR validation results revealed that all 10 ascites samples tested positive. The positive concordance rate between the immunochromatographic test strip and PCR was 70%, further corroborating the reliability of the test strip results. The colloidal gold immunochromatographic test strip developed in this experiment exhibited strong recognition ability for the target antigen in feline ascites samples, delivering accurate and reliable detection outcomes.

## DISCUSSION

FCoV infection is widespread among felids. Although most infected cats do not exhibit obvious clinical symptoms, certain FCoV variants can lead to severe diseases, such as fatal FIP (25). Therefore, controlling the viral load of FCoV in kittens is crucial for preventing the onset of FIP disease (26). Early diagnosis and prevention are essential for feline health.

Currently, there is a lack of rapid diagnostic products for FCoV, and only RT-PCR can be used for rapid detection. However, ordinary pet hospitals do not have the corresponding detection equipment, which greatly affects the discovery and diagnosis of the condition. Therefore, providing a rapid, convenient, and low-cost early rapid detection method is an urgent issue to be addressed in this field.

Choosing the N protein antibody as the recognition target for the colloidal gold strip has multiple advantages. The N protein is highly conserved in FCoV (27) and can effectively recognize multiple FCoV strains, ensuring accurate recognition of different FCoVs by the strip. The N protein is one of the most abundant viral proteins produced during the entire viral infection process (28), so the number of N proteins detected in the sample is also relatively high, which helps improve the sensitivity of detection. The presence of the N protein can be detected early in the infection (29), which is conducive to early diagnosis and intervention of coronavirus infection, thereby taking timely measures to control disease transmission and treatment. The structure of the N protein is relatively simple, and its preparation and purification are relatively easy, which helps reduce the production cost of the kit and makes it more economical and feasible for mass production and promotion. Therefore, choosing the N protein antibody as the recognition target of the colloidal gold strip is a reliable, sensitive, and cost-effective choice, providing an important tool for the rapid detection of coronavirus infection.

Based on the high conservativeness and excellent immunogenicity of the FIPV-N protein, this study successfully expressed the N protein using a prokaryotic expression system and further prepared two monoclonal antibodies, mAbs 2G7 and mAb 3H5, targeting different antigenic epitopes. The epitope sequence recognized by mAb 2G7 exhibited a homology of 97.78% among all strains, while the epitope sequence recognized by mAb 3H5 showed a homology of 98.62%. The high homology of these antigenic epitope sequences suggests the feasibility of using these two monoclonal antibodies as diagnostic reagents. In the design of the test strip, mAb 3H5 was coupled with gold nanoparticles for capturing target molecules, while the specific antibody mAb 2G7 was coated on the detection line for detecting target molecules. Compared to polyclonal antibodies, these two monoclonal antibodies, due to their high specificity and stability, are less likely to bind to non-target molecules, thus significantly improving the specificity and stability of the test strip. The high stability of monoclonal antibodies and their resistance to environmental factors ensure the long-term reliability of the test strip when using them in the construction of a double-antibody sandwich colloidal gold detection test strip. Additionally, the double-antibody sandwich structure built with two monoclonal antibodies allows for easier control of the performance and specificity of each antibody, further enhancing the performance stability and controllability of the test strip. Therefore, the double-antibody sandwich colloidal gold detection test strip constructed with two monoclonal antibodies in this study exhibits significant advantages in specificity, stability, and controllability compared to polyclonal antibodies, making it more advantageous in sample detection and improving the accuracy and reliability of the test strip.

The colloidal gold immunochromatographic test strip prepared in this study has demonstrated promising application prospects in the detection of feline ascites samples. PCR validation results showed that all samples were positive, with a concordance rate of 70% with the positive detection results of the test strip, validating the effectiveness of the test strip. However, the 30% inconsistency suggests that the performance of the test strip still needs to be improved. This inconsistency may stem from immature test strip preparation processes, unstable detection conditions, and inappropriate selection of lysis buffers. To address these issues, this study will focus on optimizing the test strip preparation process, adjusting detection conditions, and continuously selecting and optimizing lysis buffer formulations to find more effective N protein releasers that stabilize the antigenicity of N protein. As a crucial viral structural protein located within the virus, successful release of N protein is crucial for the application of colloidal gold test strips. These measures aim to further improve the accuracy and sensitivity of the test strip, perfecting and optimizing the detection method. It is worth noting that while PCR has high accuracy, its complex operation and time-consuming nature make it unsuitable for rapid screening of large sample sizes. In contrast, colloidal gold immunochromatographic test strips have greater application potential due to their simplicity, speed, and efficiency. In the future, this study will continue to optimize the test strip, striving to make it an efficient, convenient, and accurate detection tool that provides strong support for clinical diagnosis and treatment.

In summary, while the colloidal gold immunochromatographic test strip prepared in this study has demonstrated good application potential, further optimization is required to enhance its performance. Through continuous research and improvements, we aim to provide more accurate and efficient detection tools for clinical diagnosis and treatment.

### Conclusion

In this study, a panel of broad-spectrum monoclonal antibodies capable of recognizing distinct epitopes on the FCoV-N protein was prepared, and a colloidal gold immunochromatographic test strip based on the double-antibody sandwich method was successfully developed. The entire system is characterized by its simplicity in operation, cost-effectiveness, and ability to complete detection within 15 minutes, thereby significantly reducing the detection time and enhancing efficiency. The FCoV antigen rapid detection method established in this study can be employed for proactive screening of FCoV in domestic cats, effectively addressing the root cause of FIP while minimizing feline mortality.

## Data Availability

All of the data supporting the conclusions drawn in this manuscript are shown, and any reasonable request for additional data relevant to the manuscript will be met by the corresponding author.

## SUPPLEMENTAL MATERIAL

Table S1 spectrum.01830-24-s0001.docx Primers used for truncation expression of FIPV-DF-2 N.

ASM does not own the copyrights to Supplemental Material that may be linked to, or accessed through, an article. The authors have granted ASM a non-exclusive, world-wide license to publish the Supplemental Material files. Please contact the corresponding author directly for reuse.
