# Supplementary material for: A colloidal gold immunochromatographic test strip based on mAbs anti-N protein to detect feline coronavirus
Source: Microbiol Spectr. 2025 Jun 2;13(7):e01830-24. doi: 10.1128/spectrum.01830-24 (PMC12210966; doi:10.1128/spectrum.01830-24)
Supplement: Table S1 — Primers used for truncation expression of FIPV-DF-2 N. [file spectrum.01830-24-s0001.docx]

Table S1

Primers used for truncation expression of FIPV-DF-2 N

| Primer names | Primer sequence (5’-3’) | Location  (aa) | Length  (bp) |  |
| --- | --- | --- | --- | --- |
|  |  |  |  |  |
| N1-F | agattacgctcttat*ggccatggaggcc*GCCACCATGGCCACACAGGGACAAC | 1-377 | 1131 |  |
| N1-R | agatctcggtcgacc*gaattc*gGTTCGTAACCTCATCAATCATCTCAAC |  |  |  |
| N2-F | agattacgctcttat*ggccatggaggcc*GCCACCATGGCCACACAGGGACAAC | 1-110 | 330 |  |
| N2-R | agatctcggtcgacc*gaattc*gATCAATCTTGTCTTTGAATTTAGCATCAGCATG |  |  |  |
| N3-F | agattacgctcttat*ggccatggaggcc*GCCACCATGGATGCTGATGCTAAATTCAA | 100-220 | 363 |  |
| N3-R | agatctcggtcgacc*gaattc*gAGGTTTGGAATCACTACGTTCTCTAGGT |  |  |  |
| N4-F | agattacgctcttat*ggccatggaggcc*GCCACCATGGCTAAACCTAGAGAACGTAG | 210-377 | 504 |  |
| N4-R | agatctcggtcgacc*gaattc*gGTTCGTAACCTCATCAATCATCTCAAC |  |  |  |
| N5-F | agattacgctcttat*ggccatggaggcc*ATGGCCACACAGGGACAAC | 1-66 | 198 |  |
| N5-R | agatctcggtcgacc*gaattc*gTTGTTGATCCTTATTACCTATTCCTTT |  |  |  |
| N6-F | agattacgctcttat*ggccatggaggcc*TCATTCTACAACCCCATTACCCT | 34-100 | 201 |  |
| N6-R | agatctcggtcgacc*gaattc*gATGAGGTCCTGTACCTAAGAAGT |  |  |  |
| N7-F | agattacgctcttat*ggccatggaggcc*ATGGCCACACAGGGACAAC | 1-44 | 132 |  |
| N7-R | agatctcggtcgacc*gaattc*gTCCTTGTTCGAGGGTAATGGG |  |  |  |
| N8-F | agattacgctcttat*ggccatggaggcc*AACTCTCGTGGTCGGAAGA | 22-66 | 135 |  |
| N8-R | agatctcggtcgacc*gaattc*gTTGTTGATCCTTATTACCTATTCCTTT |  |  |  |
| N9-F | agattacgctcttat*ggccatggaggcc*ATGGCCACACAGGGACAAC | 1-22 | 66 |  |
| N9-R | agatctcggtcgacc*gaattc*gGTTAGAACGACCACGTCTTTT |  |  |  |
| N10-F | | agattacgctcttat*ggccatggaggcc*CCTTCCAAAAGACGTGGT | 14-34 | 63 |
| N10-R | | agatctcggtcgacc*gaattc*gCAAAGGTATATCATTATT |  |  |
| N11-F | | agattacgctcttat*ggccatggaggcc*CGTGGTCGTTCTAACTCTCGTGGTC | 18-34 | 51 |
| N11-R | | agatctcggtcgacc*gaattc*gCAAAGGTATATCATTATT |  |  |
| N12-F | | agattacgctcttat*ggccatggaggcc*CGTGGTCGTTCTAACTCTCGTGGTC | 18-32 | 45 |
| N12-R | | agatctcggtcgacc*gaattc*gGTACTTAATTTCCTGCAG |  |  |
| N13-F | | agattacgctcttat*ggccatggaggcc*CGTGGTCGTTCTAACTCTCGTGGTC | 18-30 | 39 |
| N13-R | | agatctcggtcgacc*gaattc*gAATTTCCTGCAGTGC |  |  |
| N14-F | | agattacgctcttat*ggccatggaggcc*CGTGGTCGTTCTAACTCTCGTGGTC | 18-28 | 33 |
| N14-R | | agatctcggtcgacc*gaattc*gATTCTTCCGACCACGAGA |  |  |
| N15-F | | agattacgctcttat*ggccatggaggcc*GGTCGTTCTAACTCTCGTGGTC | 19-28 | 30 |
| N15-R | | agatctcggtcgacc*gaattc*gATTCTTCCGACCACGAGA |  |  |
| N16-F | | agattacgctcttat*ggccatggaggcc*CGTGGTCGTTCTAACTCTCGTGGTC | 18-27 | 30 |
| N16-R | | agatctcggtcgacc*gaattc*gCTTCCGACCACGAGAGTTAGAACG |  |  |

Table S1 (Continued)

| Primer names | | Primer sequence (5’-3’) | Location (aa) | Length (bp) |  |
| --- | --- | --- | --- | --- | --- |
|  |  |  |  |  |  |
| N18-F | | agattacgctcttat*ggccatggaggcc*GCTGAATGTGTTCCATCAGTGTCT | | 274-377 | 312 |
| N18-R | | agatctcggtcgacc*gaattc*gGTTCGTAACCTCATCAATCATCTCAA | |  |  |
| N19-F | | agattacgctcttat*ggccatggaggcc*GCTGAATGTGTTCCATCAGTGTCTA | | 274-310 | 111 |
| N19-R | | agatctcggtcgacc*gaattc*gCTTTGGCAGGTAGTAGGTGTGAG | |  |  |
| N20-F | | agattacgctcttat*ggccatggaggcc*TCTGCTGAAGAAGCTGGTGA | | 290-327 | 114 |
| N20-R | | agatctcggtcgacc*gaattc*gTCGCTTGTAAGCGTCAATC | |  |  |
| N21-F | | agattacgctcttat*ggccatggaggcc*GGTGATCAAGTGAAAGTC | | 295-325 | 93 |
| N21-R | | agatctcggtcgacc*gaattc*gGTAAGCGTCAATCTGTTC | |  |  |
| N22-F | | agattacgctcttat*ggccatggaggcc*GGTGATCAAGTGAAAGTC | | 295-323 | 87 |
| N22-R | | agatctcggtcgacc*gaattc*gGTCAATCTGTTCTAGGAA | |  |  |
| N23-F | | agattacgctcttat*ggccatggaggcc*GATCAAGTGAAAGTCACG | | 296-323 | 84 |
| N23-R | | agatctcggtcgacc*gaattc*gGTCAATCTGTTCTAGGAA | |  |  |
| N24-F | | agattacgctcttat*ggccatggaggcc*GGTGATCAAGTGAAAGTC | | 295-322 | 84 |
| N24-R | | agatctcggtcgacc*gaattc*gAATCTGTTCTAGGAATTGACTAGT | |  |  |

Note: The lowercase letters represent the homologous arms of the pCMV-HA-DsRed vector and identify the sequence of the enzyme cutting site (with the italicized and underlined bases indicating the recognition sequence of the enzyme). In contrast, the uppercase letters denote the primer sequences for each truncated gene fragment.
